# Papillary Thyroid Carcinoma in Paediatric Age

**Published:** 2020-05-31

**Authors:** A. Garzi, M. Prestipino, E. Calabrò, R.M. Di Crescenzo, M.S. Rubino

**Affiliations:** 1 Division of Pediatric M.I.S. and Robotic Surgery University of Salerno, Italy; 2 Division of Pediatric Surgery A.O. S. Maria della Misericordia Perugia, Italy; 3 Department of Advanced Biomedical Sciences, Pathology Unit, University of Naples Federico II

**Keywords:** Thyroid, Carcinoma, Pediatric age

## Abstract

In children, differentiated thyroid carcinoma is a rare condition. Early diagnosis is not always easy, because of the lack of clinical symptoms, but it has a pivotal role in performing a correct therapeutic process.

The study describes three cases of papillary thyroid carcinoma. None of the three patients had a positive familiarity or exposure to risk factors. In two cases, the tumor occurred as a non-injurious swelling in the anterior cervical region, in the other case it occurred with a latero-cervical lymphadenopathy that had been persistent for a year. In the first two patients we made a certain diagnosis by the needle aspiration of the thyroid nodule; in the other case the diagnosis was made by surgical exeresis and histological analysis of the lymph nodes. We also performed blood chemistry and hormonal tests, neck ultrasound, chest x-ray.

The three children underwent total thyroidectomy and two of them also underwent right-sided cervical lymph node exeresis because there was the presence of metastasis.

In our experience, the best therapeutic strategy for children with differentiated thyroid carcinoma is the total thyroidectomy, followed or not by latero-cervical lymph node exeresis and radioiodiotherapy. The removal of the whole gland reduces the risk of relapse.

## I. INTRODUCTION

Thyroid carcinoma is rare in children, especially in patients under 10 years of age (1). In Europe, its incidence is estimated at around 1–2 cases per million in children aged 10–14 years compared to 70 cases per million in adults (2). The incidence rate is higher in females than in males, with a sex ratio ranging from 2:1 in children aged 0–14 years to 3,5:1 between 15–19 years (3). From the histological point of view, four major types of thyroid cancer are recognized: papillary and follicular carcinoma (differentiated carcinoma), medullary carcinoma and anaplastic carcinoma.

The aetiology of thyroid cancer is still not completely understood. Exposure to ionizing radiation may promote the development of a differentiated thyroid cancer, especially in children, probably due to increased sensitivity and proliferative activity of thyrocytes compared to adults (4).

However, medullary carcinoma correlates with some hereditary factors such as the mutation of the RET proto-oncogene and recurs in families with MEN syndrome (multiple endocrine cancers).

Follicular cancer is related to both dietary iodine deficiency and predisposing genetic factors that are not yet clear. The authors describe the diagnostic and therapeutic process of three clinical cases of patients suffering from papillary thyroid carcinoma, observed at University Hospital of Salerno.

### Case 1

Male, 14 years old. At the age of 12, an indolent tumefaction was detected in the thyroid region. In another hospital he underwent a biopsy with a histological result of papilliferous carcinoma. Then, the child underwent subtotal thyroidectomy surgery at the same site.

After 2 years, because of the appearance of some right latero-cervical tumefactions, he came to our department. At the physical examination, the right laterocervical region presented ovoid masses, arranged along the sternocleidomastoid muscle, with a well-defined contour, with an irregular anterior surface and a hard-elastic consistency, not adhering to the skin, but was united to the deep cutaneous tissues and it was painless.

An otorhinolaryngological specialist consultation revealed a hemiparesis from the right vocal cord compensated by the left chord, probably related to a injury of the right recurrent nerve. We performed a neck ultrasound (US) and the chest and skeletal x-ray which excluded pleuro-parenchymal, mediastinal and bone alterations. Also thyroid scintigraphy was performed; it showed persistence of captive tissue in the thyroid region. The “Total Body” scintigraphic examination, performed with I131, revealed two modest adsorptive thyroid residues, one at the isthmus and the other less absorbing at the level of the upper portion of the right lobe of the thyroid, with no lesions in the rest of the organism. It was decided to schedule surgery to remove the two known formations and a lymph node package at the right jugular vein below the sternocleido-mastoid muscle.

**Fig. 1:**
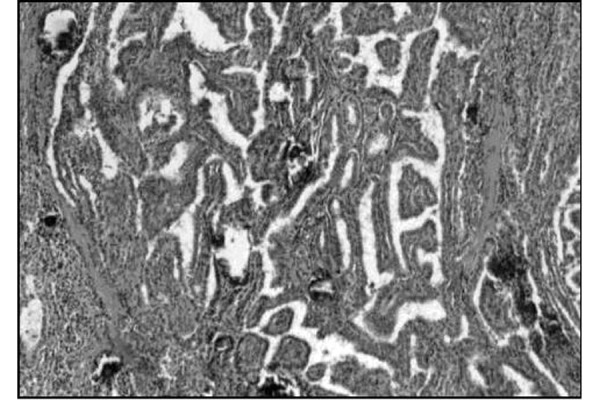
Clinical case n ° 1. Histological aspect of papillary thyroid carcinoma.

The histological examination of the removed lymph nodes confirmed neoplastic infiltration by papilliferous carcinoma (Fig. 1), while the two formations were made up of healthy thyroid tissue. The patient was discharged, on the 15th post-operative day, with iodine thyroglobulin replacement therapy.

Periodic clinical, radiological and laboratory tests performed for up to three years were within normal limits.

### Case 2

**Fig. 2:**
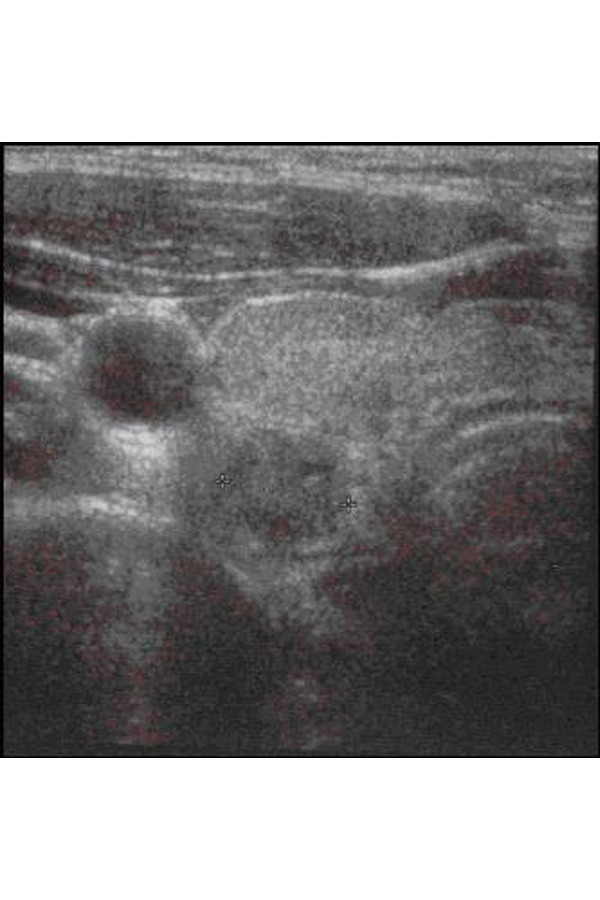
Clinical case 2. Ultrasound image of laterocervical lymph-adenomegaly, of maximum diameters up to 17x10 mm, with inhomogeneous structure

Female, 16 years old. She came to our observation for the presence of a right laterocervical tumefaction which had been persistent for a year. The three US controls performed after 1, 2 and 3 months revealed laterocervical lymph-adenomegalies with a maximum diameter of 17x10 mm, an inhomogeneous echo-structure and peripheral vascularization (Fig. 2).

The thyroid, which was evaluated during the same tests, did not present any pathological element.

At the local physical examination, we observed the presence of a right laterocervical oval tumefaction, of hard-elastic consistency, mobile on the underlying planes, slightly painful on palpation, not painful spontaneously. Because of the persistence of the clinical and diagnostic situation, it was decided to perform the surgical exeresis and biopsy of the lymph node.

Histology reported tissue infiltration of papillary differentiated thyroid carcinoma. For this reason, after 20 days, a further ultrasound examination was performed and it revealed a nodule at the right thyroid lobe of 13 mm, with microcalcifications and intralesional vascularization, associated with lymph adenomegalies in the latero-cervical site and in the thyroid lodge and small nodules size in the isthmic site and left lobe.

At this point, total thyroidectomy surgery was necessary with right laterocervical compartment resection and positioning of two drains at the level of the thyroid lodges. On the third postoperative day, the following parameters were measured: FT4, FT3, TSH, TG, anti-TG, anti-TPO, anti-TSH receptors, calcitonin; (within the normal limits). On the fourth and fifth day the two drainages were removed and on the seventh day the patient was discharged in good general condition and with replacement therapy with levothyroxine.

She subsequently performed a cycle of radioiodiotherapy. At the follow-up at 1 and 3 months the patient appeared in good conditions.

### Case 3

Female, 6 years old. At the age of 5 years, during a routine pediatric check, a nodule was noted in the right anterior region of the neck. For this reason, a thyroid ultrasound was performed, which highlighted the presence of “a single nodule at the level of the middle third level of the right thyroid lobe with a solid echo-structure, isoechogen with halo calcifications, vascularized type III, of the size of 7.8x10x14 mm “.

An ago-biopsy was also performed but, since the examination was not executed in narcosis, the removal of sufficient material for diagnosis was not possible. The blood values of anti-TPO, anti-TG, TSH, FT4 and FT3 were normal. After 2 months, the ultrasound examination showed an increased isoechogenic nodule (17x12 mm), with hypoechogenic halo and peripheral vascularization, in a right thyroid lobe increased in volume. This picture was compatible with hyperplastic nodule.

**Fig. 3:**
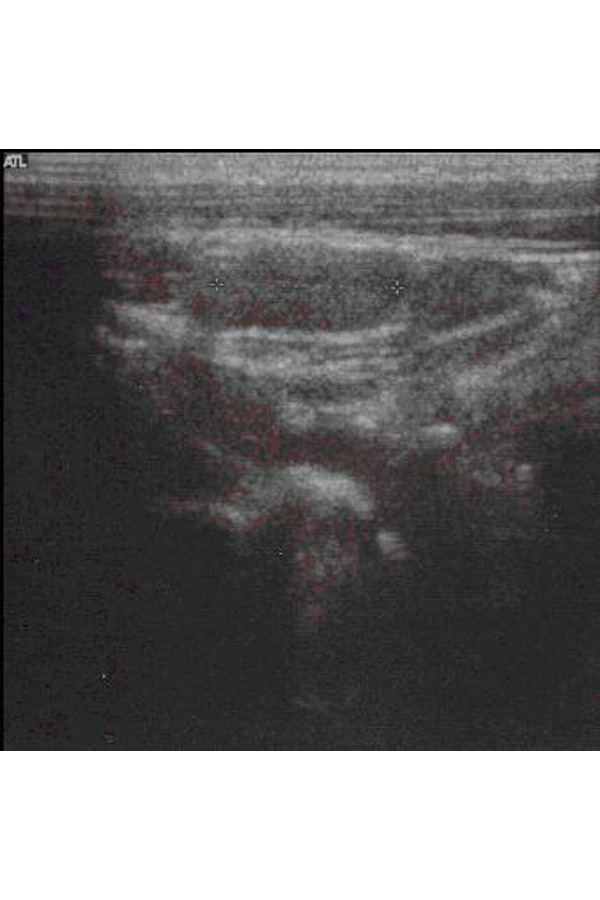
Clinical case 3. Ultrasound image of a nodule at the level of the right thyroid lobe of 21x13 mm, with internal calcifications.

At the age of 6, the patient comes to our observation in good general condition. The physical examination of the neck showed a right front rounded formation, with a smooth surface, mobile on the levels below and with swallowing, not mobile with the tongue protrusion, not adhering to the skin and not sore nor painful. A new ultrasound examination (Fig. 3) confirmed a further right thyroid nodule increased in volume (21x13 mm), with internal calcifications and peripheral vascularization, and with reactive bilateral laterocervical lymphadenopathies (maximum diameter of 15 mm on the right and 13 mm to the left).

It was therefore considered necessary to perform a new aspiration in narcosis, in order to have a clear histological diagnosis of this neoformation. The result was compatible with papilliferous carcinoma.

Total thyroidectomy surgery was performed, with positioning of two drainages in the two thyroid lodges. FT3, FT4, TSH, anti-TG, FSH were all in the normal ranges. The chest x-ray was negative. On the second day, the patient underwent thyroid replacement therapy with levothyroxine. On the third and fourth day the two drainages were removed.

The girl was discharged after a 7-day hospital stay. The child began radioiodiotherapy cycle.

## II. DISCUSSION

Differentiated thyroid carcinoma in children has a good prognosis in the majority of cases. In paediatric age, it is a rare condition and its annual incidence is about 0.5 cases/100,000 worldwide.

The affected population is very heterogeneous, and it is necessary to distinguish epidemiologically and prognostically between prepubertal children and adolescents. The 5-year survival rate is estimated 98% in children aged 0–14 and 99% in adolescents aged 15–19 (3). We considered two cases in adolescence and one in prepubescent age.

Papillary thyroid carcinoma in children differs significantly from adulthood particularly in terms of size and multifocality (5–6). Generally, children have large sizes and multifocal tumors; in our experience all cases presented an indolent mass at diagnosis (13mm in case 2 and 17x12mm in case 3).

The main features of the three cases are summarized in Table 1.

In pediatric age there is an increased incidence of lymph node metastases at diagnosis, which we observed either in the 1st case or in the 2nd case. Moreover, in the 2nd case lymph node involvement was the first sign of illness. Considering the incidence of distant metastases this is higher in pediatric age compared to adults (7% of patients in the age <20 years compared to 2% in subjects > 20 years) with a higher risk of recurrence. In fact, at 16.6 years of follow-up, recurrence occurs in 40% of patients in whom the tumor occurred at <20 years of age and in 20% of patients with 20 to 50 years at diagnosis(7).

Despite the increased incidence of relapse ( 8) and the increased number of metastases at diagnosis, the survival of children with differentiated thyroid cancer is greater than in adults. This better prognosis is certainly to be connected with a greater responsiveness of children’s functionally and metabolically active cancer cells to radioiodiotherapy and a more effective immune response of the body. In conclusion, compared to adults, in children there is a clear prevalence of the papillary type against the follicular, an element that can be linked to the child’s increased thyroid susceptibility to ionizing radiation, which is at the basis of the onset of papillary carcinoma.

Our choice to perform a radical surgery instead of the subtotal thyroidectomy in presence of lymph node involvement, arises precisely from the need to reduce the risk of recurrence. In fact, analyzing our 3 clinical cases we observed that in case 1 the subtotal thyroidectomy did not guarantee a complete removal of the tumor, which relapsed after two years.

Because of the high incidence of lymph node metastasis and the increased frequency of relapse, early diagnosis is extremely important in differentiated thyroid carcinoma in the pediatric age.

In all three cases an accurate diagnostic process, characterized by careful clinical examination, ultrasound examination and histological confirmation was performed.

US may highlight nodules with a diameter of less than 15 mm, with irregular margins and increased intranodular vascularization (9, 10).

However, in case 2 the initially US examination showed exclusively laterocervical lymphadenopathy without thyroid alterations, while a further US revealed a nodule at right thyroid lobe of 13 mm.

Finally, through the fine-needle aspiration and the subsequent histological examination of the neoformation, it is possible to have a diagnosis of certainty. This examination presents a high sensitivity, (from 94% up to 100%) (11, 12), a specificity around 74.9% and a positive predictive value of 89% (13). The fine-needle aspiration allows us to evaluate the histopathological characteristics of a thyroid nodule and directs the therapeutic choice in order to avoid unnecessary surgical treatments in healthy children.

In children with papillary thyroid carcinoma the treatment to perform is surgery. Although there is the possibility of complications in total thyroidectomy such as hypoparathyroidism (in case of ablation of the parathyroid glands) and dysphonia up to the aphonia (in case of injury of the right and left recurrent nerves) we did not find post-operative alterations.

Total thyroidectomy is associated with the removal of the lymph nodes of the thyroid lodge, especially if they are macroscopically involved (14–15). A total thyroidectomy involves subsequent life-long replacement therapy.

Controversial is the utility of laterocervical lymph nodes dissection that is mainly performed in case of certain diagnosis of tumor infiltration (16); two of our patients with lateral-cervical lymph node involvement performed the lymphadenectomy while case 3 underwent total thyroidectomy without lymph node dissection.

It is also important consider the usefulness of post-surgical radioiodine therapy, as it has a low rate of complications or side effects and has a high specificity for residual or metastatic thyroid tissues.

The association of surgery and radioiodiotherapy allows a higher selectivity on the residual cancer (5-18-19). For this reason, we subjected two patients (case 2 and 3) to post-surgical treatment with radioiodium.

## III. CONCLUSION

Differentiated thyroid carcinoma in children is a rare tumor, but not to be underestimated. The importance of early diagnosis is linked to the high frequency with which it given repetitions and relapses. However, proper treatments still allow a good survival rate and an excellent quality of life.

The most appropriate therapeutic strategy should be implemented early and includes a first surgical phase of complete ablation of the gland associated or not with exeresis of the latero-cervical lymph nodes and a second phase of radioiodiotherapy that further completes the healing of young patients.

## Figures and Tables

**Tab 1:** main features of the three cases

	Case 1	Case 2	Case 3
**Sex**	Male	Female	Female
**Age at diagnosis**	12 years old	16 years old	6 years old
**Clinical presentation**	Latero-cervical tumefactions	Latero-cervical tumefaction which had been persistent for a year	Nodule in the right anterior region of the neck
**Lymph node involvement**	Yes	Yes	No
**Medical tests**	Thyroid ultrasound, Ches t and skeletal x-ray, Thyroid scintigraphy	Thyroid ultrasound, biopsy of the lymph node.	Thyroid ultrasoud, ago-biopsy
**Surgical treatment**	-Total thyroidectomy - latero-cervical compartment resection	-Total thyroidecto my - latero-cervical compartme nt resection	-Total thyroidect omy
**Post-surgical radioiodine therapy**	No	Yes	Yes
